# Resolution of unilateral, recalcitrant dermal hypersensitivity reaction of unknown etiology with dupilumab

**DOI:** 10.1016/j.jdcr.2026.06.013

**Published:** 2026-06-16

**Authors:** Reethi Padmanabhan, Lauryn M. Falcone

**Affiliations:** a University of Pittsburgh School of Medicine, Pittsburgh, Pennsylvania; b University of Pittsburgh Medical Center, Community Medicine Inc. Dermatology, Trafford, Pennsylvania

**Keywords:** biologic therapy, dermal hypersensitivity reaction, eosinophilic dermatitis, unilateral eruption

Dermal hypersensitivity reaction (DHR) is a histopathologic diagnosis with a broad clinical presentation often with both eczematous and urticarial features.^1^ It may be triggered by infections, medications, and malignancies or can be idiopathic. Dupilumab, approved to treat both atopic dermatitis and chronic spontaneous urticaria, has been reported to improve DHR in cases with diffuse pruritus and rash.^2^^,^^3^ Here, we report a case of strikingly localized, unilateral, and recalcitrant DHR, which rapidly resolved with dupilumab.

A 32-year-old woman presented with a 1-month history of minimally scaly, erythematous macules and papules along only her left arm and hand (Fig 1). Initially, the rash resolved with a 10-day prednisone taper but recurred. She then trialed daily oral antihistamines, hydrocortisone, and switched to fragrance-free products, but these provided no relief. A midpotency topical steroid was prescribed, but she again experienced minimal relief. Initial biopsy results demonstrated perivascular dermatitis with scattered eosinophils and overlying mild acanthosis without significant spongiosis or parakeratosis. Clobetasol ointment was trialed with minimal improvement, after which topical tacrolimus ointment was prescribed on alternating days with clobetasol, along with a repeat course of prednisone taper that again provided temporary relief. Following a 2-week break from oral and topical steroids, a repeat biopsy was performed and demonstrated tight perivascular and periadnexal dermatitis with lymphocytes, histiocytes, and eosinophils, without significant epidermal changes (Fig 2).

**Fig. 1:**
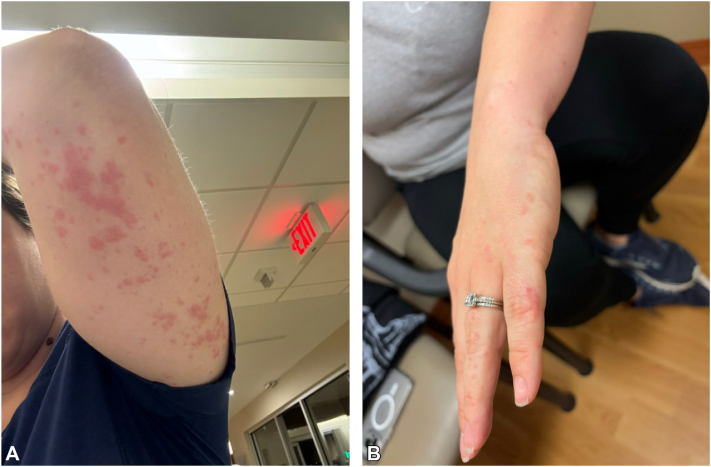
Erythematous papules coalescing into plaques on **(A)** left arm and **(B)** left superior hand and fingers.

**Fig. 2:**
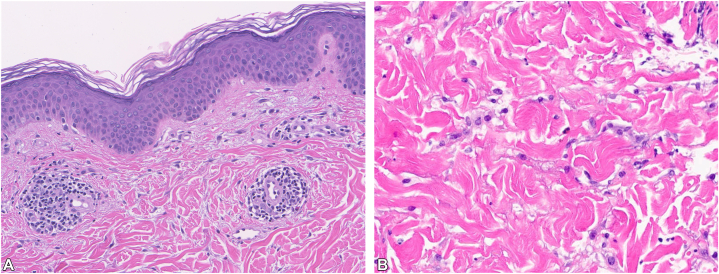
Histopathologic findings from the patient’s initial and repeat biopsy. **A,** Initial biopsy demonstrates mild acanthosis overlying a superficial and deep-dermal infiltrate of lymphocytes, histiocytes, and rare scattered eosinophils. **B,** Repeat biopsy demonstrating tight perivascular and periadnexal dermatitis with lymphocytes, histiocytes, and eosinophils. (**A** and **B,** Hematoxylin-eosin stain; original magnifications: **A,** '10; **B,** '10.)

Laboratory workup of complete blood cell count, comprehensive metabolic panel, lipid panel, hepatitis C, tuberculosis, and HIV was unremarkable. The patient used albuterol as needed for asthma for years but was otherwise healthy and denied any new medications, travel, or exposures prior to the eruption. Patch testing was also offered but declined because of the distance from the testing site. More extensive laboratory workup, such as serum IgE, thymus and activation-regulated chemokine, and *Helicobacter pylori* testing, was deferred because of insurance coverage.

A differential diagnosis including drug eruption, arthropod bite reaction, urticaria, atopic dermatitis, and viral exanthem was considered but less favored based on the patient's history, clinical appearance, and otherwise normal laboratory workup. The patient was therefore diagnosed with unilateral DHR of unknown etiology. She was then trialed on dupilumab injections at standard cutaneous dosing, given this medication’s approval for treating eczematous and urticarial dermatoses. Dupilumab provided rapid resolution of her rash within a month with sustained remission at 3, 6, and 12 months of follow-up, prompting tapering and discontinuation of the medication.

This case describes an uncommon presentation of an intractable, unilateral DHR, which completely resolved with dupilumab. The pathogenesis of DHR is not fully understood, but some subtypes represent an inflammatory response mediated by T helper 2 cells, such as type IVb reactions. Dupilumab inhibits interleukin 4 and interleukin 13 signaling, key cytokines in T helper 2 pathways, which may explain its efficacy in this case despite the absence of a classic eczematous or urticarial phenotype. The unilateral distribution raises the possibility of localized immune dysregulation or cutaneous mosaicism, although the exact mechanism remains unclear. Dupilumab is currently Food and Drug Administration approved for only 4 dermatologic conditions. This report suggests consideration of its use in other more recalcitrant dermatoses with similar histopathologic features.

## Conflicts of interest

None disclosed.
